# Mechanisms of gas transfer impairment utilizing nitric oxide following severe COVID‐19 pneumonitis

**DOI:** 10.14814/phy2.15660

**Published:** 2023-04-05

**Authors:** Leigh M. Seccombe, David Heath, Claude S. Farah, James R. Di Michiel, Elizabeth M. Veitch, Matthew J. Peters

**Affiliations:** ^1^ Thoracic Medicine, Concord Hospital Concord New South Wales Australia; ^2^ Faculty of Medicine and Health The University of Sydney Camperdown New South Wales Australia

**Keywords:** capillary blood volume, COVID‐19, diffusing capacity, gas transfer

## Abstract

Reduced carbon monoxide diffusing capacity (DL_CO_) is common after recovery from severe COVID‐19 pneumonitis. The extent to which this relates to alveolar membrane dysfunction as opposed to vascular injury is uncertain. Simultaneous measurement of nitric oxide diffusing capacity (DL_NO_) and DL_CO_ can partition gas diffusion into its two components: alveolar–capillary membrane conductance (D_mCO_) and capillary blood volume (V_C_). We sought to evaluate D_mCO_ and V_C_ in the early and later recovery periods after severe COVID‐19. Patients attended for post‐COVID‐19 clinical review and lung function testing including DL_NO_/DL_CO_. Repeat testing occurred when indicated and comparisons made using *t*‐tests. Forty‐nine (eight female) subjects (mean ± SD age: 58 ± 13, BMI: 34 ± 8) who had severe COVID‐19 pneumonitis, WHO severity classification of 6 ± 1, and prolonged (21 ± 22 days) hospital stay, were assessed 2 months (61 ± 35 days) post discharge. DL_CO_adj (*z*‐score −1.70 ± 1.49, 25/49 < lower limit of normal [LLN]) and total lung capacity (*z*‐score −1.71 ± 1.30) were both reduced. D_mCO_ and V_C_ and were reduced to a similar extent (*z*‐score −1.19 ± 1.05 and −1.41 ± 1.20, *p* = 0.4). Seventeen (one female) patients returned for repeat testing 4 months (122 ± 61 days) post discharge. In this subgroup with more impaired lung function, DL_CO_adj improved but remained below LLN (*z*‐score −3.15 ± 0.83 vs. −2.39 ± 0.86, *p* = 0.01), 5/17 improved to >LNN. D_mCO_ improved (*z*‐score −2.05 ± 0.89 vs. −1.41 ± 0.78, *p* = 0.01) but V_C_ was unchanged (*z*‐score −2.51 ± 0.55 vs. −2.29 ± 0.59, *p* = 0.16). Alveolar membrane conductance is abnormal in the earlier recovery phase following severe COVID‐19 but significantly improves. In contrast, reduced V_C_ persists. These data raise the possibility that persisting effects of acute vascular injury may contribute to gas diffusion impairment long after severe COVID‐19 pneumonitis.

## Graphical abstract

Simultaneous measurement of nitric oxide and carbon monoxide diffusing capacity (DL_NO_/DL_CO_) was used to quantify the contributions of alveolar–capillary membrane conductance and capillary blood volume (V_C_) to impaired gas diffusion following severe COVID‐19. At 2 months, both were reduced to a similar extent. Membrane conductance significantly improved at 4 months, however, V_C_ remained reduced. These novel findings suggest persisting vascular injury may contribute to physiological impairment long after severe COVID‐19 pneumonitis.

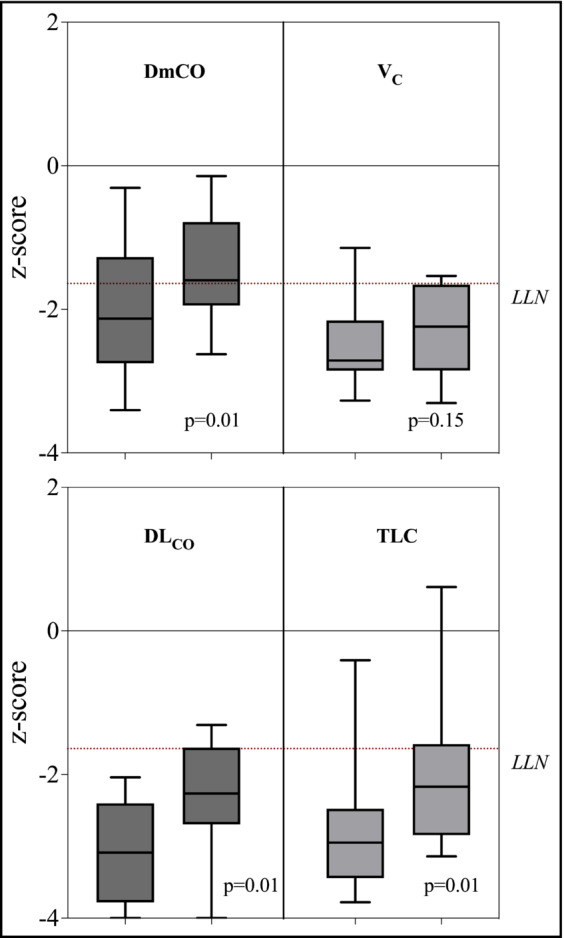

## 1 INTRODUCTION

Infection with the delta variant of COVID‐19 was frequently associated with severe respiratory failure. For this and earlier variants, impaired gas exchange, reflected in reduced carbon monoxide diffusing capacity (DL_CO_), commonly persisted after the initial phase of recovery. Cohort studies have consistently found it to be the most prevalent lung function abnormality, reduced to a greater extent than either vital capacity or total lung capacity (TLC), and related to COVID‐19 disease severity (Thomas et al., 2021; Torres‐Castro et al., 2021). This persistent abnormality could be related to alveolar–capillary membrane dysfunction or vascular injury—the latter an intriguing possibility given the frequency of pulmonary embolism seen in acute COVID‐19 and contested arguments about the importance of microthromboembolism.

Effective gas diffusion from lung to circulation requires efficient transit across the alveolar membrane that is conjoined with an effectively perfused pulmonary capillary network. Of the two tracer gases most commonly used to measure gas diffusion, CO transfer is sensitive to changes in pulmonary capillary blood volume (V_C_), whereas NO transfer is more sensitive to the function of the alveolar membrane due in part to its significantly higher reaction rate with hemoglobin (*θ*NO). Utilizing the Guenard equation (Guenard et al., 1987), the simultaneous measurement of DL_NO_ and DL_CO_ can quantify the primary components of gas diffusion: alveolar membrane conductance (D_mCO_) and V_C_ (Zavorsky et al., 2017).

The simultaneous measurement of DL_NO_/DL_CO_ is complex and not widely utilized. The available data are conflicting with some early studies reporting DL_NO_ to be reduced to a greater extent than DL_CO_ in a group with a broad range of severity post COVID‐19, suggesting a primary impairment in membrane conductance (Barisione & Brusasco, 2021; Nunez‐Fernandez et al., 2021). However, a recent study of a severe cohort reported a greater resistance in V_C_ (Noel‐Savina et al., 2021). We sought to evaluate D_mCO_ and V_C_ in patients hospitalized for severe COVID‐19 pneumonitis in the early and later recovery periods.

## 2 METHODS

### 2.1 Patient characteristics and study design

This was a prospective, cohort, observational study. The study was approved by the Sydney Local Health District Human Ethics Review Board (LNR/14/CRGH/206, 2019/ETH07887, NSW, Australia) with waiver of consent.

All patients hospitalized with severe COVID‐19 pneumonitis at Concord Hospital were invited to attend an outpatient clinical review following discharge. Complex lung function was measured in all attending patients and electronic medical record data were accessed to document clinical and biochemical data pertaining to their COVID‐19 admission. Further outpatient clinical review and repeat testing occurred when indicated by their treating physician. Patients were excluded from analysis if they had known preexisting cardiopulmonary disease, recent respiratory tract infection other than COVID‐19, significant smoking history (>10 pack/year), claustrophobia, and inability to follow instruction or meet testing criteria.

### 2.2 Lung function

Spirometry, lung volumes via plethysmography, and DL_CO_ adjusted for hemoglobin (DL_CO_adj) (Masterlab; Jaeger) (Hemocue) were performed according to the American Thoracic Society/European Respiratory Society recommendations (Graham et al., 2017, 2019; Wanger et al., 2005). Reference ranges were derived from the Global Lung Initiative (Hall et al., 2021; Quanjer et al., 2012; Stanojevic et al., 2017). Patients then performed simultaneous measurement of DL_NO_ and DL_CO_ (Hyp’ Air, Medisoft) during a 5 s breath‐hold according to ERS standards and compared to reference ranges from Zavorsky et al. (2017). The diffusion components, D_mCO_ and V_C_, were calculated using Forster's equation (Guenard et al., 1987) with finite specific *θ*NO = 4.5 mL min^−1^ mmHg^−1^ and 1/*θ*CO as per Guenard et al. (2016), as recommended by ERS standards (Zavorsky et al., 2017). The lower limit of normal (LLN) of all lung function was considered the fifth percentile, that is, at −1.64 *z*‐score.

### 2.3 Statistical analysis

The normality of distribution of all variables was assessed by the Shapiro–Wilk test and then expressed as mean ± SD. Lung function when performed at two visits only was assessed using paired, two‐tailed *t*‐tests; when performed at three visits was assessed using repeated measures ANOVA, and between groups using unpaired, two‐tailed *t*‐tests. Clinical associations with lung function data were investigated using Spearman correlation analysis. A *p* < 0.05 was considered statistically significant.

## 3 RESULTS

### 3.1 Patient characteristics

Fifty‐seven (12 female) patients presented for outpatient clinical review following COVID‐19 hospitalization, with 49 (eight female) patients (mean ± SD) age 58 ± 13 years, height 169 ± 8, and BMI 34 ± 8 kg m^−2^ meeting testing criteria. The initial clinic assessment was 61 ± 35 days (~2 months) post discharge. Patients had severe COVID‐19 pneumonitis with WHO ordinal severity classification of 5.7 ± 1.1 (Rubio‐Rivas et al., 2022) and prolonged length of hospital stay (21 ± 22 days). Maximal required fraction of inspired oxygen was 48 ± 21% and 22 (45%) patients required noninvasive ventilation or intubation during admission. Peak C‐reactive protein, ferritin, and D‐dimer during the hospitalization were elevated (146 ± 93 mg/L, 1986 ± 3704 μg/L, and 5 ± 13 mg/L, respectively). As part of their COVID‐19 therapy, 41 (84%) patients received dexamethasone, 33 (67%) received baricitinib, nine (18%) received remdesivir and two (4%) received tocilizumab. A summary of patient characteristics is presented in Table 1.

**TABLE 1:** Characteristics and lung function of 49 patients following severe COVID‐19 pneumonitis on initial respiratory review 2 months post discharge.

	All patients, 2 months post discharge, *n* = 49		
Number, female	49, 12		
Age, years	58.5 ± 12.7		
Height, cm	169.4 ± 8.2		
BMI, kg m^−2^	34.0 ± 8.1		
Hemoglobin, g/L	13.0 ± 1.47		
Days post discharge	61 ± 35		
WHO ordinal severity, 0–8	5.7 ± 1.1		
	**Absolute**	**% reference**	***z*‐score**
FEV_1_ L	2.74 ± 0.72	87.2 ± 16.8	−0.83 ± 1.19
FVC L	3.32 ± 0.92	83.1 ± 16.8	−1.15 ± 1.21
FEV_1_/FVC %	83.2 ± 7.1	—	0.66 ± 1.08
TLC L	5.07 ± 0.97	79.3 ± 15.7	−1.71 ± 1.30
FRC L	2.50 ± 0.49	78.2 ± 16.2	−1.20 ± 0.96
RV L	1.75 ± 0.38	89.7 ± 20.6	−0.46 ± 0.80
DL_CO_adj mL min^−1^ mmHg^−1^	19.0 ± 5.9	76.1 ± 20.9	−1.70 ± 1.49
K_CO_adj mL min^−1^ mmHg^−1^ L^−1^	3.98 ± 0.69	90.6 ± 15.0	—
DL_NO_ mL min^−1^ mmHg^−1^	100.8 ± 33.5	72.0 ± 19.8	−1.61 ± 1.25
D_mCO_ mL min^−1^ mmHg^−1^	97.7 ± 35.0	71.3 ± 21.5	−1.19 ± 1.05
V_C_ mL	47.3 ± 18.8	69.3 ± 24.2	−1.41 ± 1.20

Abbreviations: BMI, body mass index; DL_CO_adj, carbon monoxide diffusing capacity adjusted for hemoglobin; DL_NO_, nitric oxide diffusion capacity; D_mCO_, CO alveolar membrane conductance; FEV_1_, forced expiratory volume in 1 s; FRC, function residual capacity; FVC, forced vital capacity; K_CO_adj, DL_CO_adj adjusted for alveolar volume; RV, residual volume; TLC, total lung capacity; V_C_, capillary blood volume; WHO, World Health Organization.

Seventeen (one female) patients (33%) returned for repeat testing (Visit 2) 122 ± 61 days (~4 months) since discharge. Seven male patients returned again for a third visit (Visit 3) 232 ± 61 days (~8 months) since discharge.

### 3.2 Lung function

Lung function in all patients on the initial respiratory review 2 months post discharge is presented in Table 1. Mean DL_CO_adj (76 ± 21% reference, −1.70 ± 1.49 *z*‐score) and TLC (79 ± 16% reference, −1.71 ± 1.30 *z*‐score) were both reduced. D_mCO_ and V_C_ were reduced to a similar extent (*z*‐score −1.19 ± 1.05 and −1.41 ± 1.20, *p* = 0.4). DL_CO_adj (*r* = −0.47, *p* = 0.01) and TLC (*r* = −0.47, *p* = 0.01) were negatively correlated with length of stay. V_C_ was negatively correlated with length of stay (*r* = −0.42, *p* < 0.01). WHO severity negatively correlated with TLC only (*r* = −0.42, *p* = 0.01). Demographic and biochemical data during hospitalization did not correlate with lung function.

**FIGURE 1:**
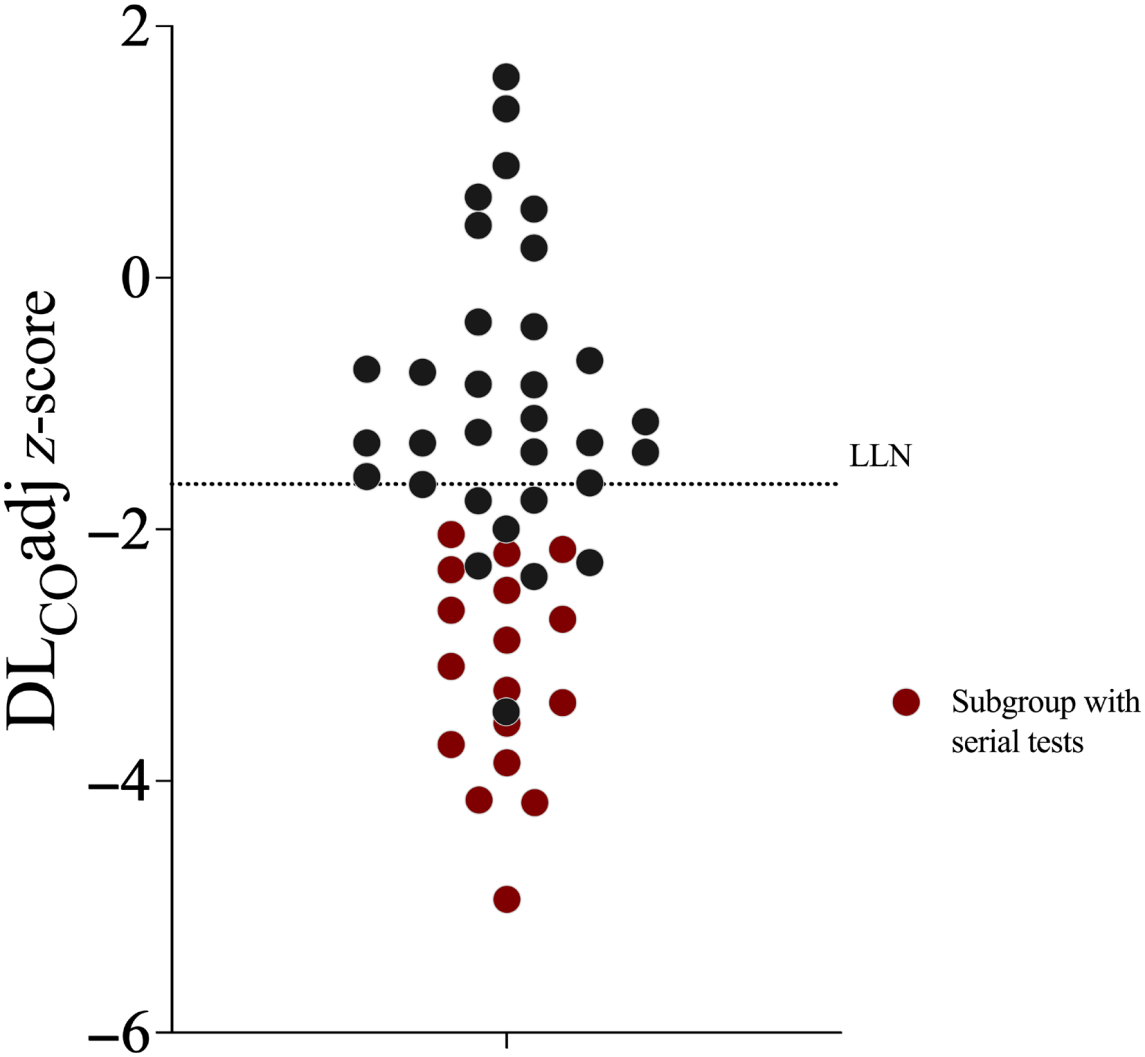
Individual *z*‐score data points of carbon monoxide diffusion capacity adjusted for hemoglobin (DL_CO_adj) on initial respiratory review in 49 patients following severe COVID‐19 pneumonitis. Red color denotes subgroup with serial testing. LLN: lower limit of normal (broken line).

**FIGURE 2:**
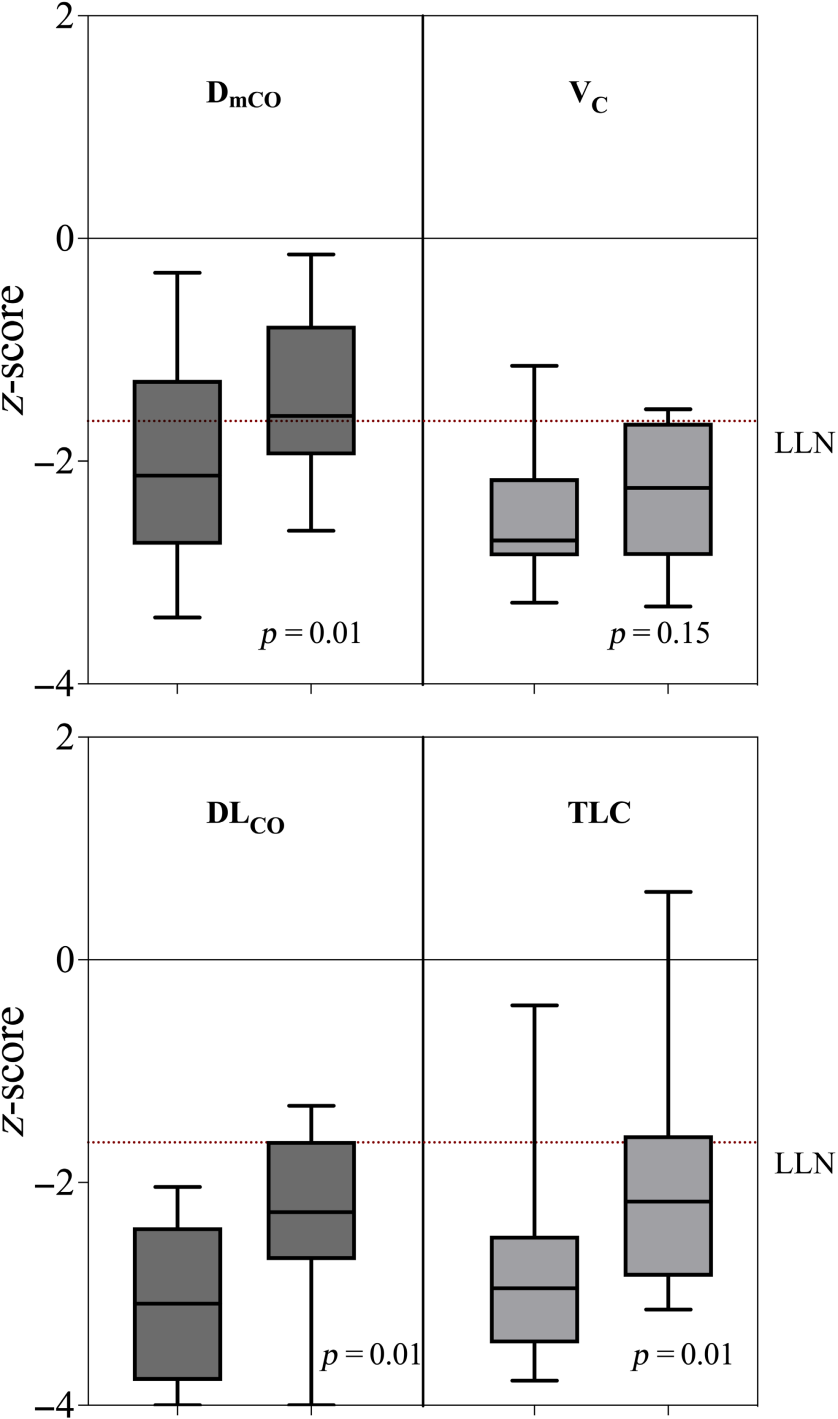
Box and whisker (95% CI) *z*‐score of alveolar membrane conductance (D_mCO_), capillary blood volume (V_C_), carbon monoxide diffusion capacity adjusted for hemoglobin (DL_CO_adj), and total lung capacity (TLC), in 17 patients at 2 and 4 months recovery from severe COVID‐19 pneumonitis. LLN: lower limit of normal (broken line). *p*‐value: paired *t*‐test.

The patient subgroup that returned for repeat visits had worse lung function than those that did not return (Table 2), WHO ordinal disease severity (6.1 ± 0.7, *p* = 0.047) and longer length of stay (29.9 ± 16.3 days, *p* = 0.01) compared to those who attended a single visit. All patients in this subgroup presented with DL_CO_adj < LLN on initial review (56.7 ± 10.0% reference, −3.15 ± 0.83 *z*‐score) (Figure 1). In this subgroup, all primary measures of lung function improved between Visit 1 and Visit 2 (Table 2). DL_CO_adj improved but remained below LLN (−3.15 ± 0.83 [Visit 1] vs. −2.39 ± 0.86 [Visit 2] *z*‐score, *p* = 0.01). D_mCO_ also improved (−2.05 ± 0.87 [Visit 1] vs. −1.41 ± 0.78 [Visit 2] *z*‐score, *p* = 0.01), however, V_C_ was unchanged (−2.51 ± 0.55 [Visit 1] vs. −2.29 ± 0.59 [Visit 2] *z*‐score, *p* = 0.16) (Figure 2). DL_NO_/DL_CO_ was at the higher range of normal (normal range: 3.8–5.8; Zavorsky et al., 2017) at Visit 1 (5.35 ± 0.25), and trended higher at Visit 2 but remained within normal range (5.42 ± 0.59) (*p* = 0.63).

**TABLE 2:** Serial lung function in a subgroup of 17 patients following severe COVID‐19 pneumonitis.

*n* = 17 (1 female)	2 months post discharge	4 months post discharge	*p*‐value (paired *t*‐test)
FEV_1_			
% reference	78.0 ± 15.9	86.4 ± 19.4	0.01*
*z*‐score	−1.42 ± 1.29	−0.83 ± 1.65	0.01*
FVC			
% reference	69.7 ± 13.0	79.9 ± 16.7	0.01*
*z*‐score	−2.08 ± 0.98	−1.32 ± 1.36	0.01*
FEV_1_/FVC			
% reference	87.6 ± 2.8	84.5 ± 4.3	0.01*
*z*‐score	1.42 ± 0.66	0.92 ± 0.94	0.01*
TLC			
% reference	66.2 ± 9.9	74.8 ± 11.9	0.01*
*z*‐score	−2.80 ± 0.81	−2.07 ± 0.94	0.01*
FRC			
% reference	67.8 ± 14.6	71.7 ± 15.0	0.01*
*z*‐score	−1.82 ± 0.96	−1.50 ± 0.81	0.01*
RV			
% reference	74.3 ± 10.5	82.0 ± 13.9	0.01*
*z*‐score	−1.02 ± 0.44	−0.72 ± 0.50	0.01*
DL_CO_adj			
% reference	56.7 ± 10.0	65.7 ± 11.3	0.01*
*z*‐score	−3.15 ± 0.83	−2.39 ± 0.86	0.01*
K_CO_adj			
% reference	84.1 ± 16.1	88.0 ± 15.6	0.02*
DL_NO_/DL_CO_			
	5.35 ± 0.25	5.42 ± 0.59	0.63
DL_NO_			
% reference	52.1 ± 12.6	61.4 ± 11.8	0.01*
*z*‐score	−2.89 ± 0.72	−2.38 ± 0.76	0.01*
D_mCO_			
% reference	52.8 ± 18.1	69.2 ± 16.6	0.01*
*z*‐score	−2.05 ± 0.87	−1.41 ± 0.78	0.01*
V_C_			
% reference	48.9 ± 11.9	52.3 ± 14.5	0.27
*z*‐score	−2.51 ± 0.55	−2.29 ± 0.59	0.15

Abbreviations: DL_CO_adj, carbon monoxide diffusing capacity adjusted for hemoglobin; DL_NO_, nitric oxide diffusion capacity; D_mCO_, CO alveolar membrane conductance; FEV_1_, forced expiratory volume in 1 s; FRC, function residual capacity; FVC, forced vital capacity; K_CO_adj, DL_CO_adj adjusted for alveolar volume; RV, residual volume; TLC, total lung capacity; V_C_; capillary blood volume.

* Met significance.

In the seven male patients that returned again for Visit 3, repeated measured identified an improvement in FVC, K_CO_adj, and D_mCO_ from 2 to 8 months following discharge (Table 3).

**TABLE 3:** Serial lung function in a subgroup of seven male patients following severe COVID‐19 pneumonitis.

*n* = 7 male	2 months post discharge	4 months post discharge	8 months post discharge	*p*‐value (repeated measures ANOVA)
Hemoglobin g/L	12.5 ± 1.4	14.1 ± 1.4	14.4 ± 1.5	0.15
FEV_1_				
% reference	86.0 ± 21.6	92.7 ± 28.7	97.7 ± 29.2	0.12
FVC				
% reference	77.0 ± 16.7	85.3 ± 24.1	91.1 ± 23.8	0.03*
FEV_1_/FVC				
% reference	87.4 ± 2.9	84.6 ± 4.7	85.3 ± 5.8	0.20
TLC^a^				
% reference	70.6 ± 16.1	78.6 ± 18.1	83.6 ± 20.0	0.16
FRC^a^				
% reference	72.2 ± 23.0	77.8 ± 21.6	77.6 ± 25.0	0.22
RV^a^				
% reference	67.6 ± 11.5	75.0 ± 10.9	82.2 ± 15.6	0.21
DL_CO_adj				
% reference	58.6 ± 9.3	66.4 ± 11.0	72.6 ± 14.3	0.16
K_CO_adj				
% reference	80.9 ± 17.8	87.6 ± 17.5	90.3 ± 21.6	0.01*
DL_NO_/DL_CO_				
	5.28 ± 0.33	5.44 ± 0.64	5.32 ± 0.64	0.81
DL_NO_				
% reference	50.9 ± 9.0	60.1 ± 9.5	65.0 ± 10.3	0.06
D_mCO_				
% reference	49.0 ± 14.9	69.4 ± 16.9	76.4 ± 18.1	0.01*
V_C_				
% reference	53.3 ± 11.3	52.0 ± 11.5	58.5 ± 19.1	0.29

Abbreviations: DL_CO_adj, carbon monoxide diffusing capacity adjusted for hemoglobin; DL_NO_, nitric oxide diffusion capacity; D_mCO_, CO alveolar membrane conductance; FEV_1_, forced expiratory volume in 1 s; FRC, function residual capacity; FVC, forced vital capacity; K_CO_adj, DL_CO_adj adjusted for alveolar volume; RV, residual volume; TLC, total lung capacity; V_C_, capillary blood volume.

^a^ *n* = 5.

* Met significance.

## 4 DISCUSSION

In this study of patients previously hospitalized for severe COVID‐19 pneumonitis, gas diffusion was reduced. In the earlier recovery phase at 2 months post discharge, there were similar reductions in both alveolar–capillary membrane conductance D_mCO_ and V_C_. In those with more severe diffusion impairment, membrane conductance significantly improved to the normal range at subsequent follow‐up near 4 months post discharge. Reduced V_C_ however persisted in this group, contributing to the ongoing impairment in gas diffusion.

These novel findings in recovery suggest vascular injury and/or persistent impairment of the pulmonary microcirculation plays an important role in the commonly observed gas diffusion impairment following severe COVID‐19. This is supported by the recent positive correlations between the extent of post COVID‐19 DL_CO_ impairment and CT markers of pulmonary perfusion (Dierckx et al., 1985; Price et al., 2022), endothelial abnormalities (Mendez et al., 2022), and reduced V_C_ (Noel‐Savina et al., 2021).

The DL_NO_/DL_CO_ ratio in our cohort was within normal limits and did not change between visits. Membrane conductance and capillary volume were similarly reduced at 2 months post infection, with an identified improvement in the former only in a more severe subgroup at 4 months. This is in contrast to two previous studies that reported greater abnormality with membrane conductance and less vascular involvement (Barisione & Brusasco, 2021; Nunez‐Fernandez et al., 2021). The differences in our findings to these previous studies may be attributed to our more severe cohort, and staged testing periods following discharge. Our initial measurements identified a similarly reduced membrane conductance and V_C_; however, we tracked a significant improvement in membrane conductance only.

There were limitations to our study. Conducting complex physiological research during the Delta and Omicron phases of the COVID‐19 epidemic was challenging and constrained by community and institutional restrictions at times. We could not test patients during the acute phase of illness, leaving us to gather insights from those who had survived, recovered sufficiently to be discharged home, and able to attend for testing at a later date. Some patients remained too unwell to attend the clinic or were unable to meet the minimum lung volume requirements for testing criteria. In contrast, because attendance at follow‐up was voluntary, we have limited insight into changes seen in those who had an excellent improvement in respiratory symptoms and did not attend. It is evident from the data (Figure 1) that there is less follow‐up information on those with more favorable lung function at the initial visit. Nevertheless, the serial measurement in the subgroup of patients with more severe disease provides a unique insight into a condition that remains poorly understood and is increasingly recognized as a significant morbidity in previously well individuals.

The derivation of D_mCO_ and V_C_ is made on the assumptions of a finite *θ*NO as considered appropriate and recommended by ERS standards (Zavorsky et al., 2017). Similar outcomes have been demonstrated in both normal and restrictive cohorts when utilizing finite versus infinite specific *θ*NO, with linear relationships between D_mCO_ and V_C_ observed in both scenarios (Barisione et al., 1985). It is recognized that technical factors relating to manufacturer variations and algorithm assumptions create large changes in calculated D_mCO_ and V_C_ (Radtke et al., 2021). This was also observed in our laboratory, and as such we manually calculated the components, and compared to reference equations as per ERS recommendations (Zavorsky et al., 2017). Utilizing a similar method to our study, Barisione et al. (1985) found good agreement with D_mCO_ and fibrotic changes in patients with idiopathic interstitial pneumonias. D_mCO_ was reduced to a greater extent than V_C_ in their cohort reflecting known parenchymal disease. Further investigation with larger and diverse cohorts are required to confirm the clinical utility of this method in clinical management.

## 5 CONCLUSION

D_mCO_ is abnormal in the earlier recovery phase of severe COVID‐19 pneumonitis but improves to a significant extent in subsequent months. In contrast, reduced V_C_ persists. Repeat testing at even longer intervals after recovery from acute illness is still required but these data raise the possibility that persisting features of acute vascular injury will contribute to physiological impairment long after severe COVID‐19 pneumonitis.

## AUTHOR CONTRIBUTIONS

*Concept and design*: Leigh M. Seccombe and Claude S. Farah. *Data collection*: Leigh M. Seccombe, James R. Di Michiel, and David Heath. *Data analysis*: Leigh M. Seccombe and David Heath. *Manuscript preparation*: Leigh M. Seccombe. *Manuscript edit and review*: All authors.

## FUNDING INFORMATION

This study was supported in part by The Jeffrey J. Pretto Memorial Research Grant.

## CONFLICT OF INTEREST STATEMENT

There are no conflicts of interest to declare.

## ETHICS STATEMENT

The study was approved with waiver of consent by the Sydney Local Health District Human Ethics Review Board (LNR/14/CRGH/206, NSW, Australia).
